# Establishment of reference interval for thyroid-stimulating hormone using electrochemiluminescence assay in a healthy adult population from Fortaleza, Brazil

**DOI:** 10.20945/2359-3997000000264

**Published:** 2020-07-17

**Authors:** Maria Helane C Gurgel, Clarisse M. M. Ponte, Rosita Fontes, Ítalo V. Rocha, Lívia A. A. Batista, Tamara C. S. Sousa, Renan M. Montenegro

**Affiliations:** 1 Universidade Federal do Ceará Programa de Pós-graduação em Ciências Médicas Faculdade de Medicina Fortaleza CE Brasil Universidade Federal do Ceará, Programa de Pós-graduação em Ciências Médicas, Faculdade de Medicina, Fortaleza, CE, Brasil; 2 Diagnósticos da América Fortaleza CE Brasil Diagnósticos da América (DASA), Endocrinologia, consultoria médica, Fortaleza, CE, Brasil; 3 Centro Universitário Christus Faculdade de Medicina Fortaleza CE Brasil Centro Universitário Christus (Unichristus), Faculdade de Medicina, Fortaleza, CE, Brasil; 4 Complexo Hospitalar Universidade Federal do Ceará Unidade de Pesquisa Clínica Fortaleza CE Brasil Complexo Hospitalar, Universidade Federal do Ceará, Unidade de Pesquisa Clínica, Fortaleza, CE, Brasil; 5 Instituto Estadual de Diabetes e Endocrinologia Luiz Capriglione Fortaleza CE Brasil Instituto Estadual de Diabetes e Endocrinologia Luiz Capriglione (IEDE), Endocrinologia, Fortaleza, CE, Brasil; 6 Universidade Federal do Ceará Programa de Pós-graduação em Saúde Coletiva Faculdade de Medicina Fortaleza CE Brasil Universidade Federal do Ceará, Programa de Pós-graduação em Saúde Coletiva, Faculdade de Medicina, Fortaleza, CE, Brasil

**Keywords:** Reference interval, thyroid stimulating hormone, reference range

## Abstract

**Objective:**

This study aimed to determine the thyroid-stimulating hormone (TSH) reference interval (RI) and to assess the influence of the use of thyroid ultrasonography (TUS) on reference individual selection from a healthy adult population in Fortaleza, Brazil.

**Subjects and methods:**

This cross-sectional study recruited patients (N = 272; age = 18-50 years) with normal thyroid function (NTF) and placed them in three groups according to their test results: NTF (n = 272; all participants), TUS (n = 170; participants who underwent thyroid US), RI (n = 124; reference individuals with normal TSH levels). TSH, FT4, TT3, TgAb, and TPOAb concentrations were determined by electrochemiluminescence assay. TUS was performed using a 7-12 MHz multifrequency linear transducer by two radiologists. The 2.5^th^ and 97.5^th^ percentiles of the distribution curve corresponded to lower and upper TSH RI levels, respectively.

**Results:**

The mean TSH level was 1.74 ± 0.96 mIU/L, and TSH range was 0.56-4.45 mIU/L. There was no difference in the TSH concentrations between men and women nor between the groups. TUS did not appear to be an essential tool for the reference group selection.

**Conclusion:**

The upper limit of TSH was comparable to the reference interval provided by the assay manufacturer (4.45 vs. 4.20 mIU/L) but the lower limit was not (0.56 vs. 0.27 mIU/L). This finding may have a clinical impact since these values may lead to the misdiagnosis of euthyroid patients with subclinical hyperthyroidism.

## INTRODUCTION

Thyroid function is regulated by a dynamic hormonal system, involving the thyroid-stimulating hormone (TSH) and thyroid hormones – free T4 (FT4) and T3 (FT3) ( 1 ). Minimal changes in free thyroid hormones levels result in significant variations in plasma TSH concentrations ( 2 , 3 ), meaning TSH levels can be used as highly sensitive indicators of thyroid function. Therefore, the precise determination of reference values – reference interval (RI) – for plasma TSH levels is crucial in clinical practice ( 4 ).

Comparing individual results with the RI is vital in medical decisions. Current guidelines on laboratory medicine recommend every clinical analysis laboratory establish its own RIs for all analytes, considering the peculiarity of the local population. The Clinical and Laboratory Standards Institute (CSLI) recommends a minimum of 120 reference individuals to determine the RI of an analyte ( 4 ); this RI would represent approximately 95% of the values found in a said population.

Pre-analytical variables and selection of healthy subjects have the most significant impact on thyroid function testing outcomes. Determination of TSH RI, for example, requires the selection of reference individuals with normal thyroid function, including the absence of symptoms, negative family history of thyroid disease, and absence of autoantibodies to thyroid antigens. Besides, the presence of normal thyroid parenchyma in thyroid ultrasonography (TUS) evaluation has been considered an indicator of normal thyroid function ( 5 , 6 ).

Despite previous recommendations, in Brazil, very few clinical laboratories determine their own RIs for different analytes, including TSH. This study aimed to determine the TSH RI and to assess the influence of the use of TUS on reference individual selection from a healthy adult population in Fortaleza, Brazil.

## SUBJECTS AND METHODS

### Study population

A cross-sectional study was conducted by the *Universidade Federal do Ceará* (UFC) and *Diagnósticos da América* (DASA). We invited healthy employees and their relatives from different hospitals and DASA laboratory collection stations distributed in all Fortaleza health districts, aged between 18 and 50 years old, of both genders.

### Study protocol

The protocol included extensive evaluation of clinical, laboratory, and imaging findings. The participants answered a self-report questionnaire on sociodemographic and medical history. Anthropometric measurements and physical examination of the neck regions were performed to assess the presence of thyroid nodules or goiter. Subsequently, they were sent for blood sample collection after 12 hours of fasting for measuring TSH, FT4, total T3 (TT3), antithyroid peroxidase antibody (TPOAb), and thyroglobulin antibody (TgAb) levels. The blood samples were collected in the morning.

The final evaluation consisted of TUS performed by two radiologists, who were thyroid experts; they used a standard model for the recording and assessment of TUS findings. Abnormal ultrasonography findings included the presence of goiter, nodule(s), and heterogeneous echo pattern.

**Figure 1:**
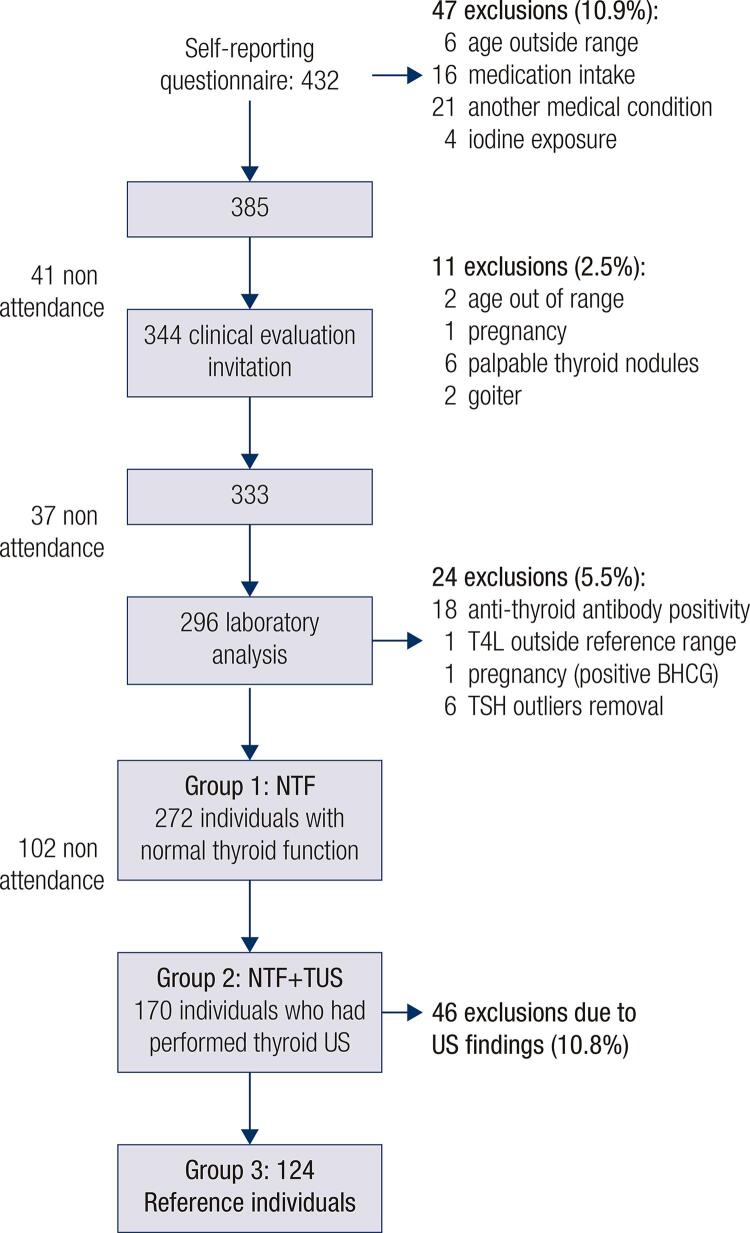
Reference individuals selection.

Initially, 432 individuals were evaluated ( Figure 1 ). The exclusion criteria were as follows: personal or family history of thyroid disease; current or previous exposure to iodine; abnormal levels of thyroid hormones; TPOAb or TgAb positivity; drug use that might affect FT4, TT3, or TSH level; chronic illness; and pregnancy.

A total of 272 individuals with normal thyroid function ([NTF]: group 1) were selected, of which 170 underwent TUS (group 2). After exclusion of individuals with abnormal TUS findings, 124 remained in the reference individual group with NTF and normal TUS (RI: group 3). To determine the RI of TSH in these groups, we adopted 2.5^th^ (lower limit) and 97.5^th^ (upper limit) percentiles of the TSH distribution curve.

### Laboratory and image evaluation

Plasma TSH concentration was determined by electrochemiluminescence immunoassay performed using modular analytics equipment by E170-Roche^**®**^ . The measurement range, defined by the lower and upper detection limit, was 0.005-100.0 mIU/L. The intra-assay coefficient of variation was 1.2%, and the inter-assay coefficient of variation was 20%. The functional sensitivity, represented by the lowest concentration of TSH that can be reproducibly measured, was 0.014 mIU/L. This method has been standardized in accordance with the Third International Standard for Thyroid-Stimulating Hormone, Human, for Immunoassay by the Expert Committee on Biological Standardization of the World Health Organization in November 2003 ( 7 ). The RI defined by the test kit was 0.270 to 4.20 mIU/L. Levels of FT4, TT3, TgAb, and TPOAb were determined by electrochemiluminescence assay (modular analytics equipment, E170-Roche^**®**^ ).

TUS was performed using a 7-12 MHz multifrequency linear transducer by Toshiba Xsario^**®**^ from Toshiba Japan Corporation. The inter-observer agreement between the radiologists was previously calibrated, showing a significant inter-observer agreement on the kappa test (k: 0.91, p < 0.001).

### Sample calculation and statistical analysis

The sample size selection for the TSH RI followed the CSLI recommendations ( 4 ) of a minimum of 120 healthy individuals. The 2.5^th^ and 97.5^th^ percentiles of the distribution curve corresponded to lower and upper TSH levels, respectively. The 95% confidence interval (95% CI) was calculated for the lower and upper TSH limits.

TSH distributions were examined using histograms and analytical methods (Kolmogorov-Smirnov and Shapiro-Wilk tests) to determine whether they were normally distributed or not. TSH values above three standard deviations (±3,0 SD) were considered outliers and were excluded. Observations judged aberrant by the researcher were also considered outliers ( 4 ).

### Ethical aspects

The Ethical Committee of the University of Ceará Hospital approved this study, and written informed consent was obtained from all subjects. This study was conducted as per the recommendations of the Declaration of Helsinki.

## RESULTS

**Figure 2:**
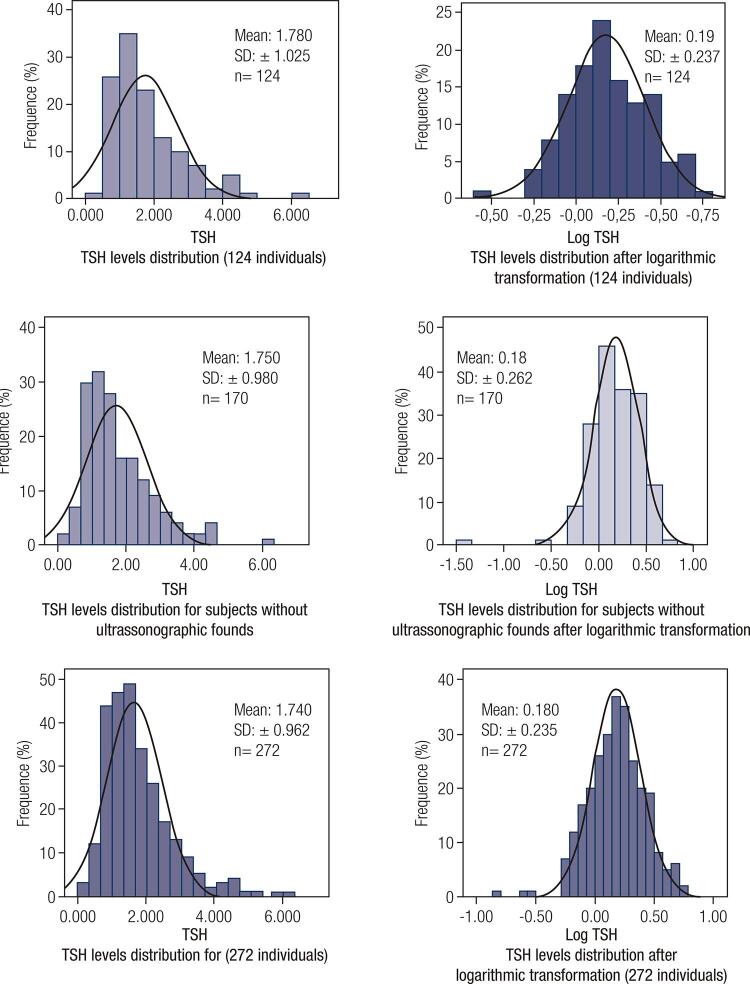
TSH distribution curves.

The baseline characteristics and TSH evaluation results of the groups are presented in Table 1 , and TSH distribution curves are demonstrated in Figure 2 . Three individuals with outlier TSH values (mUI/mL: 6.06 [SD: +3.5], 8.58 [SD: +5,7], and 11.19 [SD: +7,9]) were excluded; one with TSH value 0.03 mUI/mL was excluded by clinical judgment.

**Table 1:** Basal characteristics and TSH evaluation (mean, reference interval and confidence interval) in three groups.

Variable	Group 1 (n: 272)	Group 2 (n: 170)	Group 3 (n: 124)	P
Age, years (mean ± SD)	34.5 ± 11.2	35.8 ± 11.1	33.1 ± 10.3	0.205
Female gender, n (%)	183 (67.2)	126 (74.1)	83 (66.4)	0.304
TSH, mUI/L (mean ± SD)	1.74 ± 0.96 ♂ 1.78 ± 0.81 ♀ 1.65 ± 0.87 p: 0.074*	1.75 ± 0.98 ♂ 1.85 ± 0.99 ♀ 1.71 ± 0.95 p: 0.630*	1.78 ± 1.02 ♂ 1.78 ± 1.10 ♀ 1.74 ± 1.01 p: 0.557*	0.920
TSH reference interval (mIU/L)	0.56 – 4.44	0.58 – 4.43	0.56 – 4.45	NA
TSH reference interval (confidence interval)	2.5^th^: 0.49 – 0.62 97.5^th^: 3.94 – 4.89	2.5^th^: 0.56 – 0.67 97.5^th^: 3.97 – 4.51	2.5^th^: 0.34 – 0.65 97.5^th^: 4.01 – 5.87	NA

Group 1: participants with NTF; group 2: participants with NTF who had performed TUS; group 3: reference individuals with NTF and TUS without abnormalities SD: standard deviation. Significant p-value if < 0.05. *between male (♂) and female (♀) genders. NA: not applicable.

In the NTF group (n = 272), female gender showed predominance (183, 67.2%), and the mean age was 34.5 ± 11.2 years. The mean TSH concentration was 1.74 ± 0.96 mIU/L and RI was 0.56-4.44 mIU/L.

In the TUS group (n = 170), there were 126 (74.1%) women, and the mean age was 35.8 ± 11.1 years. In this group, 46 (27.0%) showed abnormal TUS findings: thyroid nodules (n = 32), parenchyma echotexture abnormalities (n = 8), nodules associated with abnormalities of parenchymal echotexture (n = 3), and nodules related to goiter (n = 3). The mean TSH concentration was 1.750 ± 0.98 mIU/L and RI was 0.58-4.43 mIU/L.

In the RI group (n = 124), there were 83 (66,4%) women, and the mean age was 33.1 ± 10.3 years. The mean TSH level was 1.780 ± 1.025 mIU/L and RI was 0.56-4.45 mIU/L.

## DISCUSSION

To the best of our knowledge, this is the first study designed to establish the TSH RI in Fortaleza. We included individuals without thyroid disease, confirmed by clinical evaluation, laboratory analysis (FT4, TT3, TgAb, and TPOAb), and TUS. The TSH RI for this healthy adult reference group was 0.56-4.45 mIU/L.

The TSH concentrations could be influenced by several conditions, including age, ethnicity, genetic, gender, iodine nutritional status, presence of thyroid autoantibodies, thyroid disease, medication, nonthyroidal illness, and assay type ( 8 - 10 ). Besides, genetic influences play a major role in maintaining the hypothalamic-pituitary-thyroid axis ( 11 , 12 ).

These aspects reinforce the importance of the determination of the TSH reference range in different populations, according to geographic region and age group. However, in many the clinical analysis laboratories, the TSH reference range used is the one provided by the assay manufacturer, and these values are not always validated for the local population.

Early signs of autoimmune thyroid disease may manifest as thyroid echotexture alterations ( 13 ). In order to ensure that individuals with early thyroid disease were not included in our RI sample, we performed TUS and excluded all cases with any abnormalities. Mean and reference range values of TSH for the TUS and NTF groups – based on clinical and laboratory findings – were practically identical. Therefore, TUS did not appear to be an essential tool for the identification of healthy thyroid function, as described in previous studies ( 14 , 15 ). In fact, the CLSI guidelines state that TUS routine examination is not required for reference group selection ( 4 ) – this optimizes research costs and also saves the patient from undergoing unnecessary examinations.

In our study, the TSH upper limit value was comparable to that provided by the assay manufacturer (4.45 *vs.* 4.20 mIU/L), but the lower limit was different (0.56 *vs.* 0.27 mIU/L). This finding is of clinical impact; these values could lead to the misdiagnosis of euthyroid patients with subclinical hyperthyroidism, requiring further investigation or treatment.

The reasons why the TSH lower limit was higher in our sample are not clear. The National Survey for Evaluation of the Impact of Salt Iodination evaluated the panorama of the nutritional status of iodine in Brazil. This study collected data on iodine intake in 18 Brazilian states, including Ceará; excess iodine consumption was found in the northeast regions of Brazil ( 16 ). As previously demonstrated, high iodine intake could be associated with an increase in TSH levels ( 17 , 18 ). However, we cannot confirm this association because we did not evaluate the iodine levels in this study.

Although the TSH lower limit was not comparable to that provided by kit used in our study. The TSH range found in our sample was comparable to the data previously reported, especially when we analyzed the samples according to the instructions of the assay manufacturer. A recent review evaluated the TSH RI of four different immunoassays for a healthy adult population. The TSH lower limit ranged from 0.51 to 0.63 mIU/L and upper limit ranged from 3.60 to 4.31 mIU/L ( 19 ). In this survey, the Roche^®^ assay’s TSH range was from 0.60 to 4.31 mIU/L, comparable to our data.

In Brazil, other studies evaluated the TSH RI. In Belo Horizonte, Rosario and cols. (2010) found a different TSH RI of 0.43-3.24 mIU/L in adults aged 18 to 60 years (chemiluminescent assay; Immulite 2000 platform – Diagnostic Products Corporation^®^) ( 20 ). Later, in 2014, the same authors found a TSH RI of 0.2 to 4.62 mIU/L (chemiluminescent assay; Immulite 2000 platform – Diagnostic Products Corporation^®^), in an older population aged 70-85 years ( 21 ). Fontes and cols. (2013) found a TSH RI of 0.4-4.3 mIU/L in the age group of 20-59 years (electrochemiluminescence immunoassay; Roche Modular Analytics^®^ E170 – Roche Diagnostics) ( 22 ).

At the global level, the National Health and Nutrition Examination Survey (NHANES III) showed a TSH range of 0.45-4.12 mIU/L in US populations aged 12 years and older (measured by chemiluminescence immunometric assay – Nichols Institute Diagnostics) ( 23 ). In this survey, 13,344 individuals with no family or personal history of thyroid disease, goiter, thyroid laboratory abnormality, and use of interfering medication but with positive TgAb and TPOAb were examined. Recent European studies showed a TSH RI of 0.44 to 4.13 mIU/L in women aged between 19 and 70 years (electrochemiluminescence method – Roche Diagnostics^®^) ( 24 ) and of 0.65 to 5.39 mIU/L in women aged between 20 and 69 years (chemiluminescent immunoassay – Architect i2000 platform, Abbott Laboratories^®^) ( 25 ).

Recently, the International Federation of Clinical Chemistry (IFCC) Committee for Standardization of Thyroid Function Tests has been making efforts to harmonize and, if possible, standardize TSH measurements to achieve uniform reference values among the different assays provided by in vitro diagnostic test manufacturers. Thienpont and cols. (2017) evaluated 14 different TSH immunoassays subjected to standardized recalibration procedures. The authors performed a multi-assay method comparison study with clinical serum samples and found a TSH RI of 0.56 to 4.27 mIU/L. However, they emphasized that the RI presented in their report cannot be seen as the endpoint and should not be widely extrapolated. They suggest that, at this time, clinical laboratories should continue to determine their RI values following accepted consensus standards, such as those of the IFCC, the National Academy of Clinical Biochemistry, and CLSI ( 26 ).

However, in practice, very few laboratories adopt these recommendations owing to the inherent difficulties in selecting an appropriate reference population for all analytes in clinical practice. Thus, the use of indirect methods is an alternative for determining reference values ( 4 ). Indirect approaches are those performed using laboratory results usually collected for routine clinical care. They are faster and cheaper than direct methods and the RIs are usually determined by statistical methods based on distribution of the data, rather than requiring assessment of all individual results in the database ( 27 ).

Finally, we emphasize that normal limits for serum TSH vary greatly depending on method used to determinate the RI ( 28 ). We also highlight that when using methods based on non-parametric statistics (percentiles), 5% of euthyroid individuals will have values outside this range. Using clinical judgment to interpret the thyroid function test results is mandatory. Rather than absolute numbers, the RI values should be evaluated individually, taking into account all possible factors that could interfere with the results ( 29 ).

We observed dominance of the female gender in our study sample, which could be a limitation. Women tend to have higher TSH concentrations than men, but this increase is associated with positive thyroid peroxidase antibody status ( 6 ), a fact not observed in our study. Besides, the mean TSH levels between men and women were comparable, indicating that there is no need to determine the TSH RIs in groups subdivided by gender ( 4 ). Other possible limitations are the absence of race, smoking, and iodine sufficiency status evaluation.

In conclusion, the TSH range for this healthy adult population was 0.56 to 4.45 mIU/L. These results are partially comparable to the TSH RI provided by the assay manufacturer and by IFCC Committee for Standardization of Thyroid Function Tests. Our data also corroborate the CLSI recommendation that is not necessary to perform TUS evaluation for reference group selection. These findings should encourage more laboratories to apply CLSI recommendations in the determination of RIs for analytes in their reports, providing validated information for their specific populations.
